# Livestock-Associated, Antibiotic-Resistant *Staphylococcus aureus* Nasal Carriage and Recent Skin and Soft Tissue Infection among Industrial Hog Operation Workers

**DOI:** 10.1371/journal.pone.0165713

**Published:** 2016-11-16

**Authors:** Maya Nadimpalli, Jill R. Stewart, Elizabeth Pierce, Nora Pisanic, David C. Love, Devon Hall, Jesper Larsen, Karen C. Carroll, Tsigereda Tekle, Trish M. Perl, Christopher D. Heaney

**Affiliations:** 1 Department of Environmental Sciences & Engineering, Gillings School of Global Public Health, University of North Carolina at Chapel Hill, North Carolina, United States of America; 2 Department of Environmental Health & Engineering, Johns Hopkins Bloomberg School of Public Health, Baltimore, Maryland, United States of America; 3 Johns Hopkins Center for a Livable Future, Johns Hopkins University, Baltimore, Maryland, United States of America; 4 Rural Empowerment Association for Community Help, Warsaw, North Carolina, United States of America; 5 Microbiology and Infection Control, Statens Serum Institut, Copenhagen, Denmark; 6 Division of Medical Microbiology, Johns Hopkins University School of Medicine, Baltimore, Maryland, United States of America; 7 Microbiology Laboratory, Johns Hopkins Hospital, Baltimore, Maryland, United States of America; 8 Division of Infectious Diseases, Department of Medicine, Johns Hopkins University School of Medicine, Baltimore, Maryland, United States of America; 9 Department of Epidemiology, Johns Hopkins Bloomberg School of Public Health, Baltimore, Maryland, United States of America; Kent State University, UNITED STATES

## Abstract

Swine production work is a risk factor for nasal carriage of livestock-associated (LA-) *Staphylococcus aureus* and also for skin and soft tissue infection (SSTI). However, whether LA-*S*. *aureus* nasal carriage is associated with increased risk of SSTI remains unclear. We aimed to examine *S*. *aureus* nasal carriage and recent (≤3 months prior to enrollment) SSTI symptoms among industrial hog operation (IHO) workers and their household contacts. IHO workers and their household contacts provided a nasal swab and responded to a questionnaire assessing self-reported personal and occupational exposures and recent SSTI symptoms. Nasal swabs were analyzed for *S*. *aureus*, including methicillin-resistant *S*. *aureus* (MRSA), multidrug-resistant-*S*. *aureus* (MDRSA), absence of *scn* (livestock association), and *spa* type. *S*. *aureus* with at least one indicator of LA was observed among 19% of 103 IHO workers and 6% of 80 household members. Prevalence of recent SSTI was 6% among IHO workers and 11% among 54 minor household members (0/26 adult household members reported SSTI). Among IHO workers, nasal carriers of MDRSA and *scn*-negative *S*. *aureus* were 8.8 (95% CI: 1.8, 43.9) and 5.1 (95% CI: 1.2, 22.2) times as likely to report recent SSTI as non-carriers, respectively. In one household, both an IHO worker and child reported recent SSTI and carried the same *S*. *aureus spa* type (t4976) intranasally. Prevalence of *scn*-negative *S*. *aureus* (PR: 5.0, 95% CI: 1.2, 21.4) was elevated among IHO workers who reported never versus always wearing a face mask at work. Although few SSTI were reported, this study of IHO workers and their household contacts is the first to characterize a relation between nasal carriage of antibiotic-resistant LA-*S*. *aureus* and SSTI. The direction and temporality of this relation and IHO workers’ use of face masks to prevent nasal carriage of these bacteria warrant further investigation.

## Introduction

Over the past decade, animal-adapted strains of antibiotic-resistant *Staphylococcus aureus* have emerged globally among food-producing animals, as well as among people who have contact with food-producing animals [1]. These *S*. *aureus*, which include methicillin-resistant (MRSA) and multidrug-resistant *S*. *aureus* (MDRSA) strains, are commonly referred to as livestock-associated (LA-) *S*. *aureus*. LA-*S*. *aureus* may spread from animals to humans through direct contact and through contact with animal dander, particles from decomposing waste [2], and aerosols generated by animal activity (e.g. movement, coughing, and/or sneezing) [3]. Clonal complex (CC) 398 is the most widely described strain, although CC9 is increasingly reported in Asia [4,5] and the United States [6,7]. Nasal carriage and infection with LA-*S*. *aureus* has increased among individuals without livestock exposure in several European Union countries, suggesting that human-to-human transmission may now be occurring in the community, albeit at a lower rate than typical community-associated *S*. *aureus* CCs [8–10].

Despite high prevalence of LA-*S*. *aureus* nasal carriage among individuals occupationally-exposed to livestock [1] and increasing prevalence among some non-exposed populations [8,11], it remains unclear whether LA*-S*. *aureus* nasal carriage is a risk factor for skin and soft tissue infection (SSTI). Nasal carriage of hospital and community-associated *S*. *aureus*, which occurs among 20–40% of the general United States population, is an established risk factor for infection in the clinical setting [12]. However, LA-*S*. *aureus* appear to differ from hospital and community-associated strains in that they typically lack genetic factors associated with human infection, including Panton-Valentine leukocidin- (PVL-) encoding genes, enterotoxin-producing genes, and human immune evasion complex (e.g., *scn*) genes [13–15]. Their capacity for human-to-human transmission also appears to be lower than many widespread community- and hospital-associated *S*. *aureus* CCs [16,17]. Most LA-*S*. *aureus* infections present as SSTI, which are of concern to IHO workers who can experience lacerations, abrasions, and other skin injuries through their daily work activities [18,19]. Associations between LA-*S*. *aureus* nasal carriage and SSTI are difficult to investigate through studies in clinical settings because many livestock workers lack regular access to medical care [20], and because nasal screening for *S*. *aureus* is not typically conducted for clinical SSTI cases [21,22]. To date, prospective cohort studies of healthy volunteer hog production workers have not identified risk factors for SSTI other than livestock exposure [21,23,24]. Identifying an association between SSTI and nasal carriage, which can be managed through decolonization interventions [25], could help efforts to prevent *S*. *aureus* exposure and associated infections among livestock workers and their household and community contacts.

In the present study, we assessed nasal carriage of LA-*S*. *aureus* among industrial hog operation (IHO) workers and their household members in North Carolina and estimated associations with self-reported SSTI symptoms. Using data from the baseline enrollment visit of a four-month repeated-measures study of *S*. *aureus* nasal carriage, we examined: (a) baseline prevalence and distribution of *S*. *aureus* nasal carriage patterns; (b) associations between *S*. *aureus* nasal carriage patterns and reports of recent (in the three months prior to the baseline visit) SSTI symptoms; and (c) associations between *S*. *aureus* nasal carriage patterns and IHO work exposures.

## Materials and Methods

### Data Collection

Data were collected between October 2013 and February 2014 by community organizers from the Rural Empowerment Association for Community Help (REACH) with researchers from the Johns Hopkins Bloomberg School of Public Health (JHSPH) and the Gillings School of Global Public Health at the University of North Carolina at Chapel Hill (UNC). Community organizers recruited volunteer livestock workers who fit the following inclusion criteria: currently worked at an industrial hog operation (IHO), resided in North Carolina, could speak English or Spanish, and were at least 18 years of age. Only individuals employed in production of live pigs were considered IHO workers for the purposes of this study; individuals employed in other types of food animal production or meat processing (e.g. slaughterhouses, processing plants, rendering facilities) were not included in the study. Up to three individuals living in the same household as an IHO worker were eligible to participate if they were at least seven years old and spoke English or Spanish. Because enrolled participants were encouraged to refer IHO workers in their social networks to participate, it was not possible to determine the total number of IHO workers who learned about this study, or the percent who were not interested in contacting community organizers for further information about participating. Before participating, adults provided written informed consent. Written parental permission and informed assent were collected for participants seven to 17 years of age (hereafter referred to as “minors”). The JHSPH Institutional Review Board (IRB) approved this study (IRB00004608). The UNC Non-Biomedical IRB approved reliance on the JHSPH IRB.

Each participant attended a baseline enrollment session at the REACH office or in a community meeting space (e.g. a local church). Each enrollment session lasted between 2–3 hours. Participants responded to a baseline questionnaire which assessed demographic information, household characteristics, work activities, risk factors for exposure to *S*. *aureus*, and symptoms of SSTI and doctor-diagnosed *S*. *aureus* infection during the three months prior to the enrollment session. Participants also provided a self-collected BBL CultureSwab (BD, Sparks, MD) from both of their anterior nares, under supervision and using illustrated diagrams and verbal instruction. Nasal swabs were self-collected rather than collected by investigators based on prior experience in a repeated-measures *S*. *aureus* nasal carriage study in which participants demonstrated consistent and repeatable nasal swabbing technique [6]. Self-collected nasal swabs can be as effective in recovering pathogens from the anterior nares as investigator-collected swabs in a supervised setting [6,26–28]. Participants were provided with a monetary incentive after completing study tasks (approved by the JHSPH IRB).

### Detection of *S*. *aureus* and MRSA

Baseline swabs were transported to UNC at 4°C within 12 hours of collection. A trip blank was included with each shipment to confirm lack of contamination during transport. Within three days of arrival, each swab was clipped into one ml of phosphate-buffered saline and vortexed for 60 seconds. One hundred μL of this eluate was plate spread on CHROMagar™ Staph aureus (BD, Franklin Lakes, NJ) (CSA) while the remaining eluate and swab were refrigerated at 4°C. After incubation at 37°C for 24 hours, at least two colonies with morphology characteristic of *S*. *aureus* were streaked on quadrants of a new CSA plate. If direct plating did not yield colonies with *S*. *aureus* morphology, swabs and remaining PBS were enriched overnight at 37°C in 10 mL Mueller-Hinton broth containing 6.5% NaCl. A loopful (~10 μL) of Mueller-Hinton broth was streaked onto both Baird Parker and CSA plates to increase sensitivity of detection [29], and incubated at 37°C for 24 hours. Up to two colonies with morphology characteristic of *S*. *aureus* on either media were then streaked on quadrants of a new CSA plate. In total, presumptive *S*. *aureus* colonies were isolated within two to four days of nasal swab collection. Presumptive colonies were archived at -80°C in brain heart infusion broth with 15% (w/v) glycerol.

A crude DNA extraction was performed on each isolate using a protocol adapted from Reischl *et al*. [30]. Multiplex PCR was used to amplify the *spa*, *scn*, *mec*A, and *mec*C genes [31]. Strain LGA251 was used as an extraction and PCR control for *spa* and *mec*C, while a clinical MRSA isolate was used as an extraction and PCR control for *spa*, *scn*, and *mec*A. Sterile water was used as a negative control. PCR products were visualized on 2% agarose gels stained with ethidium bromide. Colonies positive for *spa*, a *S*. *aureus*-specific gene, were classified as *S*. *aureus*. Singleplex PCR was used to evaluate the presence of *pvl*-encoding genes among all *S*. *aureus* isolates [32].

All isolates were characterized by *spa* typing using the Ridom StaphType software and the Ridom SpaServer (http://spa.ridom.de/index.shtml) [33]. *S*. *aureus* CCs, including CC398 and CC9, are traditionally inferred through multi-locus sequence typing (MLST), which we did not perform. However, *spa* types associated with CC398 and CC9 have been described by other studies in which both MLST and *spa* typing were performed [15,34,35]. Thus, we assigned putative CC398 or CC9 to isolates in this study based on *spa type*, the literature, and the Ridom SpaServer, as others have done [6,36].

### Assessment of antibiotic susceptibility

One isolate from each *S*. *aureus*-positive nasal swab was assessed for susceptibility to a panel of antibiotics (S1 Table), using the Phoenix Automated Microbiology System (BD Diagnostic Systems, Sparks, MD). Testing was completed by the Clinical Microbiology Laboratory at the Johns Hopkins Hospital. Isolates for testing were shipped from UNC to JHU between January and February, 2014. MRSA were defined as *S*. *aureus* isolates resistant to cefoxitin and positive for either *mec*A or *mec*C [37]. MDRSA were defined as *S*. *aureus* isolates resistant to three or more classes of antibiotics. MRSA isolates meeting the definition of MDRSA were classified as multidrug-resistant MRSA. However, MRSA and MDRSA did not necessarily overlap by these definitions.

### Indicators of livestock association

There is currently no consensus marker for LA-*S*. *aureus*. We examined four indicators of livestock association among *S*. *aureus* isolates: strain type CC398, strain type CC9, absence of *scn*, and tetracycline resistance. We have used CC398, absence of *scn*, and tetracycline resistance as indicators of livestock association in previous work [6,38]. CC9 has been identified as a marker of LA-*S*. *aureus* among livestock workers in Asia [39], and has been observed among livestock herds and people in contact with these herds in the Midwestern US [7], and in the same geographic region as the present study [6,38].

### Definition of recent SSTI outcome

We created the main infection outcome variable, “any symptoms of *S*. *aureus* SSTI in the past three months,” as “Yes”, “No”, or “missing” based on participants’ responses to the baseline questionnaire. This variable was coded “Yes” for participants who reported “Yes, in the past three months” to any of the following: doctor-diagnosed *S*. *aureus* infection; skin boil; pus-filled abscess; red, painful, swollen skin bump or pimple; or spider bite that was itchy and oozes liquid; “No” for participants who reported “No” to all of the above; or as “Missing.” Prior to responding, participants were shown pictures of *S*. *aureus* SSTI with each of these manifestations (courtesy of the Centers for Disease Control and Prevention [CDC] and Dr. Tara Smith, Kent State University). Hereafter, we refer to this outcome as “recent SSTI.”

### Statistical analysis

We compared the distributions of potential individual (e.g. use of antibiotics, participation in contact sports) and household risk factors (e.g. number of household members, pets, home located on a hog operation) for *S*. *aureus* nasal carriage between workers and household members. For analyses in which prevalence of *S*. *aureus* nasal carriage was evaluated as an outcome, results for adult and minor household members were combined for sample size considerations. We calculated the prevalence of nasal carriage of *S*. *aureus*, MRSA, and MDRSA among workers and their household members, including livestock-associated classifications of the above.

We used log-binomial regression models to estimate crude prevalence ratios (PR) and 95% confidence intervals (CI) for associations between participant type (worker versus household member) and *S*. *aureus* nasal carriage outcomes, *S*. *aureus* nasal carriage outcomes and recent SSTI, and occupational exposures and *S*. *aureus* nasal carriage outcomes. We used generalized estimating equations with an exchangeable correlation matrix to account for the non-independence of observations within-household [40]. Individual and household-level risk factors that were associated with both participant type and nasal carriage patterns were included in log binomial regression models where sample size allowed. However, adjusted PRs are not presented because inclusion of these covariates did not change the magnitude or precision of effect estimates. All analyses were performed using SAS 9.3 (Cary, NC). An anonymized (de-identified) dataset has been included in the Supplemental Materials showing sufficient variables to reproduce the analysis (S1 Appendix).

## Results

### Participant and household characteristics

One hundred eighty-three participants, comprising 103 workers (56%), 26 adult household members (14%), and 54 minor children (30%) completed a baseline questionnaire and provided a baseline nasal swab (Table 1). All but two participants identified as Hispanic or African-American. Traditional risk factors for nasal carriage of antibiotic-resistant *S*. *aureus* were uncommon among IHO workers and adult household members (e.g. hospitalization, use of antibiotics, use of a gym or workout facility, and/or playing contact sports within the past three months; among adult household members, working in a medical facility was also uncommon). Among minors, recently playing contact sports (36%) and using a gym or workout facility (55%) were more common, but using antibiotics was uncommon (6%) (Table 1).

**Table 1:** Baseline characteristics of 183 industrial hog operation workers and household members participating in a cohort study of *S*. *aureus* nasal carriage in North Carolina, 2013–2014.

	Overall	Workers	Adult household members	Minor household members
	N (%)^a^	N (%)^a^	N (%)^a^	N (%)^a^
Participants	183 (100)	103 (56)	26 (14)	54 (30)
Age in years, mean (SD)	30 (16)	39 (11)	38 (15)	11 (3)
Female	94 (52)	47 (46)	19 (73)	28 (53)
Race/ethnicity				
Hispanic	161 (89)	89 (89)	23 (88)	49 (92)
African-American	17 (9)	12 (12)	2 (8)	3 (6)
Caucasian	2 (1)	0 (0)	1 (4)	1 (2)
Education				
K-5th grade	-	-	-	28 (54)
c6-8th grade	-	-	-	17 (33)
9-11th grade	-	-	-	7 (13)
<High school^b^	107 (60)	48 (48)	7 (27)	-
≥High school^b^	72 (40)	53 (52)	19 (73)	-
Contact sports				
≤3 month ago	31 (17)	12 (12)	0 (0)	19 (36)
>3 month ago	151 (83)	91 (88)	26 (100)	34 (64)
Missing	1	0	0	1
Used gym or workout facility				
≤3 month ago	40 (22)	10 (10)	1 (4)	29 (55)
>3 month ago	142 (78)	93 (90)	25 (96)	24 (45)
Missing	1	0	0	1
Used antibiotics				
≤3 month ago	13 (7)	8 (8)	3 (12)	3 (6)
>3 month ago	165 (93)	91 (92)	23 (88)	51 (94)
Missing	5	4	0	1
Hospitalized				
≤3 month ago	5 (3)	2 (2)	2 (8)	1 (2)
>3 month ago	178 (97)	100 (98)	23 (92)	53 (98)
Missing	2	1	1	0

^a^ Totals for each characteristic may not sum to the total number of participants due to missing information.

^b^ “Overall” column includes results from minors.

In total, the 183 participants comprised 81 households (S2 Table). Most households comprised three to five individuals (73%) and 45% reported having children under seven in the home. Over half reported having no health insurance (58%). Care from a private doctor was most common (44%) but 32% reported seeking care at an urgent care or emergency department for medical care (S2 Table).

### Prevalence of *S*. *aureus*, MRSA, and MDRSA

Forty-five of 103 workers (44%) and 31 of 80 household members (39%) carried *S*. *aureus* at baseline and MRSA was observed in only one worker (Table 2). Twenty-one workers (20%) and eight household members (10%) carried MDRSA (Table 2). *S*. *aureus* carried by IHO workers exhibited a larger diversity of antibiotic resistance patterns (10 distinct patterns observed) than *S*. *aureus* carried by their household members (five patterns observed) (S3 Table).

**Table 2:** Prevalence and distribution of characteristics of baseline *S*. *aureus* nasal carriage among 183 industrial hog operation workers and household members in North Carolina, 2013–2014.^a^

	Overall	Workers	Household members	PR (95% CI)^b^
	N (%)	N (%)	N (%)	
Participants	183 (100)	103 (100)	80 (100)	
*S*. *aureus*	76 (42)	45 (44)	31 (39)	1.1 (0.8, 1.5)
MRSA	1 (1)	1 (1)	0	—
MDRSA	29 (16)	21 (20)	8 (10)	2.0 (1.0, 4.0)
*scn*-negative				
*S*. *aureus*	25 (14)	20 (19)	5 (6)	2.9 (1.3, 6.5)
MRSA	1 (1)	1 (1)	0	—
MDRSA	18 (10)	14 (14)	4 (5)	2.6 (1.0, 6.7)
tetracycline-resistant				
*S*. *aureus*	21 (12)	17 (17)	4 (5)	3.3 (1.3, 8.5)
MRSA	1 (1)	1 (1)	0 (0)	—
MDRSA	15 (8)	13 (13)	2 (3)	5.2 (1.1, 23.8)
CC398^c^				
*S*. *aureus*	10 (6)	6 (6)	4 (5)	1.2 (0.3, 4.2)
MRSA	1 (1)	1 (1)	0 (0)	—
MDRSA	8 (4)	6 (6)	2 (3)	2.0 (0.5, 12.0)
CC9^d^				
*S*. *aureus*	9 (5)	9 (9)	0	—
MRSA	0	0	0	—
MDRSA	6 (3)	6 (6)	0	—
*scn*-negative, tetracycline-resistant				
*S*. *aureus*	16 (9)	13 (13)	3 (4)	3.3 (1.1, 10.3)
MRSA	1 (1)	1 (1)	0	—
MDRSA	13 (7)	11 (11)	2 (3)	4.4 (1.0, 20.3)

*Note*. MRSA = methicillin-resistant *S*. *aureus*. MDRSA = multidrug-resistant *S*. *aureus*. PR = prevalence ratio.

^a^ All *S*. *aureus* isolates with phenotypic resistance to methicillin (characterized by resistance to cefoxitin) were *mec*A positive.

^b^ “Household Members” is referent category.

^c^ All *S*. *aureus* CC398 isolates from workers were tetracycline-resistant and *scn*-negative. Three of four *S*. *aureus* CC398 isolates from household members were tetracycline-resistant and *scn*-negative. All MRSA CC398 and MDRSA CC398 isolates were tetracycline-resistant and *scn*-negative.

^d^ All *S*. *aureus* CC9 isolates were *scn*-negative. Four of nine *S*. *aureus* CC9 were tetracycline-resistant. Three of six MDRSA CC9 were tetracycline-resistant.

We observed elevated prevalence of MDRSA (PR: 2.0, 95% CI: 1.0, 4.0), *scn*-negative *S*. *aureus* (PR: 2.9, 95% CI: 1.3, 6.5), tetracycline-resistant *S*. *aureus* (PR: 3.3, 95% CI: 1.3, 8.5), tetracycline-resistant MDRSA (PR: 5.2, 95% CI: 1.1, 23.8), and *scn*-negative, tetracycline-resistant *S*. *aureus* (PR: 3.3, 95% CI: 1.1, 10.3) among workers compared to household members (Table 2). Putative *S*. *aureus* CC9 was only observed among workers (9/103) but putative *S*. *aureus* CC398, including CC398 with additional indicators of livestock association, was observed among both workers and their household members (Table 2). We did not observe *pvl*-encoding genes among any *S*. *aureus* isolates in this study.

### Prevalence of and risk factors for recent SSTI

Six of 103 IHO workers (6%) reported recent SSTI (Table 3). Three IHO workers reported a swollen skin bump, two reported a skin boil, and one reported a spider bite that was itchy. One of the IHO workers with a swollen skin bump reported this symptom was a doctor-diagnosed *S*. *aureus* infection. Six of 80 household members (8%) reported recent SSTI, and all of these household members were minors (6/54; 11%) (Table 3). Three minors (10, 11, and 12 years old) reported a swollen skin bump and two minors (7 and 8 years old) reported a spider bite that was itchy. One minor reported a doctor-diagnosed *S*. *aureus* infection, but did not describe its presentation.

**Table 3:** Association of baseline *S*. *aureus* nasal carriage patterns with recent skin and soft tissue infection (SSTI) among industrial hog operation workers and their child household members in North Carolina, 2013–2014.^a^

	Overall			IHO worker			Child^b^ household member		
	Total N^c^	Recent SSTI^b^ N (%)	PR (95% CI)	Total N^c^	Recent SSTI^b^ N (%)	PR (95% CI)	Total N^c^	Recent SSTI^b^ N (%)	PR (95% CI)
*S*. *aureus*									
Carrier	72	9 (13)	4.5 (1.4, 14.9)	42	5 (12)	6.8 (0.9, 53.0)	25	4 (16)	2.2 (0.4, 11.2)
Non-carrier	104	3 (3)	ref	57	1 (2)	ref	27	2 (7)	ref
MDRSA									
Carrier	25	4 (16)	3.1 (1.2, 7.8)	18	4 (22)	8.8 (1.8, 43.9)	5	0	—
Non-carrier	151	8 (5)	ref	81	2 (2)	ref	47	6 (13)	ref
*scn*-negative *S*. *aureus*									
Carrier	22	3 (14)	2.4 (0.5, 10.7)	17	3 (18)	5.1 (1.2, 22.2)	2	0	—
Non-carrier	154	9 (6)	ref	82	3 (4)	ref	50	6 (12)	ref
tetracycline-resistant *S*. *aureus*									
Carrier	18	2 (11)	1.6 (0.5, 4.9)	14	2 (14)	3.0 (0.6, 13.8)	1	0	—
Non-carrier	158	10 (6)	ref	85	4 (5)	ref	51	6 (12)	ref

*Note*. MDRSA = multidrug-resistant *S*. *aureus*. SSTI = skin and soft tissue infection. PR = prevalence ratio. CI = confidence interval. Ref = referent category for regression with categorical predictor variable.

^a^ No adult household member reported symptoms of a *S*. *aureus* infection at baseline. Child household member participants were between 7 and <18 years of age.

^b^ SSTI comprises individuals who reported “Yes, in the past three months” to any of the following: doctor-diagnosed *S*. *aureus* infection; skin boil; pus-filled abscess; red, painful, swollen skin bump; or spider bite that is itchy. Participants were shown pictures of *S*. *aureus* infections with each of these presentations prior to answering this question.

^c^ Totals do not sum to N = 183 (Overall), N = 103 (IHO worker), or N = 54 (Child household members) because some participants did not report whether or not they had recent SSTI.

Among all participants, we observed that those who carried *S*. *aureus* (PR: 4.5, 95% CI: 1.4, 14.9) and MDRSA (PR: 3.1, 95% CI: 1.2, 7.8) (Table 3) intranasally were more likely to report a recent SSTI. Among IHO workers, we observed a higher prevalence of recent SSTI among individuals carrying versus not carrying MDRSA (PR: 8.8, 95% CI: 1.8, 43.9) and *scn*-negative *S*. *aureus* (PR: 5.1, 95% CI: 1.2, 22.2); associations between recent SSTI and nasal carriage of *S*. *aureus* and tetracycline-resistant *S*. *aureus* were positive but not statistically significant (Table 3).

Characteristics of *S*. *aureus* isolates recovered from the anterior nares of workers and minors who reported recent SSTI are described in Table 4. One IHO worker-minor pair in the same household who reported recent SSTI were *S*. *aureus* nasal carriage positive and carried the same *S*. *aureus spa* type (t4976), although the minor’s isolate was tetracycline-susceptible (Table 4). One additional IHO worker-minor pair in the same household who reported a doctor-diagnosed *S*. *aureus* infection were *S*. *aureus*-nasal carriage negative. None of the six IHO workers who reported recent SSTI carried *S*. *aureus* CC398 but two carried *scn*-negative *S*. *aureus* CC9, one carried *scn*-negative, tetracycline-resistant t002, and one carried tetracycline-resistant t4976 (Table 4).

**Table 4:** Characteristics of *S*. *aureus* recovered from the anterior nares of industrial hog operation workers and minors who reported recent skin and soft tissue infection (SSTI) in North Carolina, 2013–2014.^a^

Household ID	Participant Type	*S*. *aureus* or MDRSA	*spa* type	Characteristics of livestock association			
				*scn*-negative	tet-resistant	CC398	CC9
A	Worker	MDRSA	t4976		**x**		
	Minor	*S*. *aureus*	t4976				
B	Worker	MDRSA	t002	**x**	**x**		
C	Worker	MDRSA	t337	**x**			**x**
D	Worker	*S*. *aureus*	t3446	**x**			**x**
E	Worker^b^	MDRSA	t008				
F	Minor	*S*. *aureus*	t688				
G	Minor	*S*. *aureus*	t233				
H	Minor	*S*. *aureus*	t031				

*Note*: SSTI = skin and soft tissue infection. MDRSA = multidrug-resistant *S*. *aureus*.

^a^ Six workers and six minors reported recent symptoms of SSTI. Five of six workers and four of six minors carried *S*. *aureus* in their nares at baseline. One worker-minor pair who reported recent SSTI lived in the same household but both did not carry *S*. *aureus* in their nares at baseline.

^b^ Worker’s recent SSTI was doctor-diagnosed as a *S*. *aureus* infection.

### Occupational risk factors for nasal carriage

Among the 103 participating IHO workers, working between 51–60 hours per week was most common (40/99) (S4 Table). Almost all workers (96%) reported always wearing boots or other foot protection while at work, but always wearing gloves (86%), and always wearing long sleeves and pants or coveralls (86%) were somewhat less common. Only 37% of IHO workers reported always wearing a face mask at work. A higher nasal carriage prevalence of MDRSA (PR: 3.9, 95% CI: 1.0, 15.2), s*cn*-negative *S*. *aureus* (PR: 5.0, 95% CI: 1.2, 21.4), and tetracycline-resistant *S*. *aureus* (PR: 4.1, 95% CI: 0.9, 17.7) was observed among workers who reported never wearing a face mask versus those who reported always wearing a face mask at work (Table 5). Examination of statistical associations between IHO workers’ occupational exposures and recent SSTI was limited due to small numbers. But we did observe increasing prevalence of recent SSTI as IHO workers’ reported frequency of face mask usage decreased (S4 Table).

**Table 5:** Associations between frequency of protective face mask usage and nasal carriage of MDRSA, *scn*-negative *S*. *aureus*, *and* tetracycline-resistant *S*. *aureus* among industrial hog operation workers in North Carolina, 2013–2014.

	MDRSA			*scn*-negative *S*. *aureus*		tetracycline-resistant *S*. *aureus*	
	N^a^ (%)	N (%)	PR (95% CI)^b^	N (%)	PR (95% CI)^b^	N (%)	PR (95% CI)^b^
Frequency of mask use							
Never	18 (17)	6 (6)	3.9 (1.0, 15.2)	4 (4)	5.0 (1.2, 21.4)	4 (4)	4.1 (0.9, 17.7)
Sometimes	45 (44)	11 (11)	0.7 (0.3, 1.8)	11 (11)	1.6 (0.5, 5.8)	9	0.9 (0.3, 2.5)
Always	37 (36)	2 (2)	Ref	3 (3)	ref	2 (2)	ref

*Note*. MDRSA = multidrug-resistant *S*. *aureus*. PR = prevalence ratio. CI = confidence interval. ref = referent category for regression with categorical predictor variable.

^a^ Total does not sum to N = 103 because some workers did not report frequency of mask use.

^b^ PR estimated using log-binomial regression model using a generalized estimating equation with an exchangeable correlation matrix to account for the non-independence of observations within households. Nasal carriage was modeled as a binary outcome.

## Discussion

In this study population of IHO workers and their household contacts, we found that nasal carriage of *S*. *aureus* was associated with recent symptoms of SSTI. Among IHO workers specifically, we observed positive associations between nasal carriage of each *S*. *aureus*-related outcome we examined (*S*. *aureus*, MDRSA, *scn*-negative *S*. *aureus*, tetracycline-resistant *S*. *aureus*) and recent SSTI. Only associations with MDRSA and *scn*-negative *S*. *aureus* were statistically significant; however, the direction and similar magnitude of these associations indicates that nasal carriage may be associated with SSTI outcomes in this study population. Overall, the twelve participants who reported recent SSTI included two IHO worker-minor pairs; one of these worker-minor pairs reported that a doctor had confirmed their SSTI symptoms as a *S*. *aureus* infection. This is one of the first reports in the United States of an association between nasal carriage of livestock-associated *S*. *aureus* and SSTI among individuals with frequent and intensive exposure to industrial hog production, as well as of SSTI prevalence among their minor child household members. This finding warrants further investigation to assess the directionality of the relation between *S*. *aureus* nasal carriage and SSTI in this population.

Few studies have surveyed SSTI among individuals employed in livestock production. In this cross-sectional analysis, we observed a SSTI prevalence of 6.6% (12/183). A retrospective analysis of reported injuries among pork meatpacking and poultry processing workers from 2004–2009 determined average annual SSTI prevalence to be 33% and 12%, respectively [41]. A cross-sectional online and paper-based survey of US pork producers identified a self-reported MRSA infection prevalence of 5/135 (3.7%) [42]. In one other published study of SSTI among hog farm owners and operators that we are aware of, Wardyn *et al*. observed an incidence of 6.6 cases/1,000 person-months [7]. The present study differed from Wardyn *et al*. in several ways. First, Wardyn *et al*. only examined farm owners and operators, for whom exposure to animals and the confinement barn environment may be less intensive than for hired laborers whose job tasks primarily involve direct contact with livestock. Second, participants in Wardyn *et al*.’s study were primarily Caucasian (98.9%), while participants in this study were primarily Hispanic (89%). Hispanic livestock production workers are believed to significantly underreport occupational injuries compared to Caucasian workers [43]. Third, Wardyn *et al*. ascertained *S*. *aureus* infections using prospectively-collected skin swabs, while we only assessed SSTI through participants’ recall of recent symptoms. Participants’ recall of SSTI symptoms may have included infections prior to the three months before enrollment, skin afflictions that were not related to a bacterial infection, and/or SSTIs with a bacterial etiology other than *S*. *aureus*. In order to minimize the possibility of reporting errors, we showed participants pictures of *S*. *aureus* SSTI (provided by the CDC and Dr. Tara Smith, Kent State University) prior to asking them to report SSTI symptoms. In our study, any potential misclassification due to recall was non-differential with respect to *S*. *aureus* nasal carriage status since participants were blinded to their lab result at enrollment. However, whether recent SSTI preceded or was a consequence of participants’ *S*. *aureus* nasal carriage could not be investigated with our study design. Fourth, Wardyn *et al*. did not describe *S*. *aureus* nasal carriage status among individuals who reported SSTI. Thus, any association between antecedent *S*. *aureus* nasal carriage and SSTI in their study population is not known. The results presented here provide some of the first insights into the association between nasal carriage of *S*. *aureus* with multiple indicators of livestock association and prevalence of SSTI among IHO workers and their household contacts in the United States.

Because we asked participants about SSTI during the three months prior to and including the enrollment session, we were unable to assess whether the *S*. *aureus* present in participants' nares were the same strains responsible for recent SSTI. However, we observed associations between nasal carriage of *S*. *aureus*, MDRSA, and *S*. *aureus* with indicators of livestock association (absence of *scn*) and self-reported SSTI. Additionally, risk factors for community-associated and healthcare-associated antibiotic-resistant *S*. *aureus* infection (e.g. recent use of antibiotics, playing contact sports, recent use of a gym) were uncommon among IHO workers and their household members. Some risk factors were more common among minors (specifically, playing contact sports and recent use of a gym).

S5 Table depicts the frequency and distribution of *S*. *aureus spa* types observed among workers and household members. Fifty-nine of the 81 households enrolled in this study contained more than one study participant. Ten of these households contained at least two individuals who shared the same *S*. *aureus spa* type at baseline (S6 Table). Seven of these ten were worker-household member pairs, two of ten were worker-worker pairs, and one of ten was a household member-household member pair. Shared *spa* types included: t094, t233, t337, t4976, t5739, t645, t659, t701 and t7226. *S*. *aureus* strains that were concordant between workers and their household members generally did not have characteristics of livestock association (CC398, CC9, tetracycline resistance or absence of *scn*). One worker-household member pair shared a tetracycline-resistant *spa* type (t701) and one worker-worker pair shared a *S*. *aureus spa* type associated with CC9 (t337), which was also *scn*-negative. One worker-household member pair shared the same *spa* type (t4976), but only the worker carried *S*. *aureus* with an indicator of livestock association (tetracycline resistance).

Although ten of 59 households contained participants who shared the same *S*. *aureus spa* type, we observed little evidence of household transmission of livestock-associated *S*. *aureus*. Other studies have detected *S*. *aureus* CC398 transmission between occupationally-exposed individuals (i.e. livestock veterinarians) and their household members [44]. We may not have observed transmission of livestock-associated *S*. *aureus* in this study population because it does not occur, or because of limitations in our study design. First, we examined transmission events in the context of a cross-sectional analysis. Since transmission events may be infrequent [16], a longitudinal study design might be better suited to capture transmission events. Further, we only examined one *S*. *aureus* isolate per nasal swab for *spa* type and indicators of livestock association. Since a portion (~7%) of *S*. *aureus*-colonized individuals carries multiple strain types simultaneously [45], it is possible we missed some instances of concordance. Finally, we assessed transmission events based on shared *spa* types between household members. However, recent whole genome sequencing studies have demonstrated that shared *spa* types may not indicate true transmission events; both false positives and false negatives are possible [46,47].

There were several limitations to our assessment of *S*. *aureus*-related carriage outcomes. First, because we directly plated nasal swabs, our sensitivity to detect MRSA may have been lower than if we used an antibiotic-supplemented enrichment broth [48]. However, MRSA prevalence among IHO workers in this study (1%) was comparable to a previous study of IHO workers we conducted in this same region (3%) [38], in which an antibiotic-supplemented enrichment broth was used. Second, since ~7% of *S*. *aureus*-colonized individuals carry multiple *S*. *aureus* strains simultaneously[45], as mentioned previously, we may not have captured the true distribution of *S*. *aureus*-related outcomes in this population. Third, we only assessed *S*. *aureus* nasal carriage to maximize participant acceptability. However, *S*. *aureus* colonization has been documented at additional body sites [49]. It is possible that we underestimated *S*. *aureus* carriage by not swabbing other body sites. However, it is likely that such underestimation would have been non-differential with respect to the outcome (i.e., similar among individuals with and without recent SSTI), which could have had an attenuating influence on our PR (95% CI) estimates. Other common *S*. *aureus* body colonization sites of IHO workers (e.g. axillary, inguinal, and rectal areas) are more likely to be protected in the IHO environment than the anterior nares. Thus, the possibility that we greatly underestimated *S*. *aureus* body carriage is likely to be low. Fourth, IHO workers and their household members volunteered to participate in this study; participants were not randomly selected from an enumerated population (e.g. employee roster/records). Thus, it is unclear how generalizable our findings are to all livestock workers in North Carolina or the United States. Nevertheless, the potential for exposure to and infection with antibiotic-resistant *S*. *aureus* among the estimated 292,000 livestock workers employed in the United States in 2012 [50–52] merits further investigation. Based on the demographics of our study population (>90% Hispanic; <50% health insurance) IHO workers in NC may be particularly vulnerable, as they may not have access to healthcare systems and thus may be difficult to capture via medical records-based or passive surveillance studies.

Additional research is needed to establish the direction and temporality of the association between nasal carriage of livestock-associated, antibiotic-resistant *S*. *aureus* and SSTI. In this study population, we observed that nasal carriage of *S*. *aureus*, including MDRSA, and *scn*-negative *S*. *aureus*, was more common among individuals who reported recent symptoms of SSTI. This association is in accordance with previously observed associations between *S*. *aureus* nasal carriage and infection in clinical settings [12], and provides some of the first evidence in the United States of a potential link between nasal carriage of livestock-associated *S*. *aureus* (a modifiable potential risk factor for infection) and SSTI. Because few cases of SSTI were reported, future studies should examine this association in a larger cohort and over time to assess its repeatability and directionality. Overall, this study adds to the growing body of evidence suggesting that individuals exposed to livestock-associated, antibiotic-resistant *S*. *aureus*, MRSA, and MDRSA via hog production work in the United States could be at risk for SSTI.

## Supporting Information

S1 Appendix Analysis dataset for baseline study of 183 industrial hog operations workers and household members in North Carolina, 2013–2014. (XLSX)

S1 Table Antibiotics used for susceptibility testing of *S*. *aureus* isolates. (DOCX)

S2 Table Baseline characteristics of 81 households participating in a cohort study of *S*. *aureus* nasal carriage in North Carolina, 2013–2014. (DOCX)

S3 Table Antibiotic resistance patterns of *S*. *aureus* recovered from the anterior nares of industrial hog operation workers and household members in North Carolina, 2013–2014. (DOCX)

S4 Table Summary of industrial hog operation worker exposures and recent SSTI in past three months in North Carolina, 2013–2014. (DOCX)

S5 Table Distribution of *S*. *aureus spa* types recovered from the anterior nares of industrial hog operation workers and household members in North Carolina, 2013–2014. (DOCX)

S6 Table Characteristics of *S*. *aureus spa* types shared within participant households in North Carolina, 2013–2014. (DOCX)
