# Supplementary material for: Livestock-Associated, Antibiotic-Resistant Staphylococcus aureus Nasal Carriage and Recent Skin and Soft Tissue Infection among Industrial Hog Operation Workers
Source: PLoS One. 2016 Nov 16;11(11):e0165713. doi: 10.1371/journal.pone.0165713 (PMC5112983; doi:10.1371/journal.pone.0165713)
Supplement: S1 Table — (DOCX) [file pone.0165713.s002.docx]

S1 Table**.** Antibiotics used for susceptibility testing of *S. aureus* isolates.

| Antibiotic class | Antibiotic tested | Resistance MIC Breakpoints (≥μg/mL) |
| --- | --- | --- |
| aminoglycoside | gentamicin | 16 |
| β-lactam | ampicillin | 0.5 |
|  | oxacillin | 4 |
|  | penicillin | 0.25 |
| cephamycin | cefoxitin | 8 |
| fluoroquinolone | moxifloxacin | 2 |
| glycopeptide | vancomycin | 16 |
| lincosamide | clindamycin | 4 |
| lipopeptide | daptomycin | 1 |
| macrolide | erythromycin | 8 |
| nitrofuran | nitrofurantoin | 128 |
| oxazolidione | linezolid | 8 |
| rifamycin | rifampin | 4 |
| streptogramin | quinupristin/dalfopristin | 4 |
| sulfonamide/trimethoprim | sulfamethoxazole/ trimethoprim | 4/76 |
| tetracycline | tetracycline | 16 |
|  | minocycline | 16 |
