# Supplementary material for: Livestock-Associated, Antibiotic-Resistant Staphylococcus aureus Nasal Carriage and Recent Skin and Soft Tissue Infection among Industrial Hog Operation Workers
Source: PLoS One. 2016 Nov 16;11(11):e0165713. doi: 10.1371/journal.pone.0165713 (PMC5112983; doi:10.1371/journal.pone.0165713)
Supplement: S2 Table — (DOCX) [file pone.0165713.s003.docx]

S2 Table. Baseline characteristics of 81 households participating in a cohort study of *S. aureus* nasal carriage in North Carolina, 2013-2014.

|  | N^a^ (%) |
| --- | --- |
| Total number of participating households | 81 |
| Study participants per household |  |
| 1 | 22 (27) |
| 2 | 21 (26) |
| 3 | 33 (41) |
| 4 | 5 (6) |
| Individuals living in household |  |
| <3 | 11 (15) |
| 3-5 | 53 (73) |
| ≥6 | 9 (12) |
| Children <7 years old living in household | 33 (45) |
| Occupations of other household members |  |
| Industrial livestock operation | 15 (19) |
| Pasture-based livestock operation | 4 (5) |
| Meat processing plant | 2 (3) |
| Wastewater treatment plant | 2 (3) |
| Medical facility or health clinic | 3 (4) |
| Any pets inside home | 14 (47) |
| Animals raised on property at home | 13 (16) |
| Hogs | 8 (10) |
| Chickens | 3 (4) |
| Turkeys | 1 (1) |
| Cows | 2 (3) |
| Horses | 1 (1) |
| Household's source of health insurance^b^ |  |
| No health insurance | 47 (58) |
| Company health insurance | 30 (37) |
| Private health insurance | 8 (10) |
| Public health insurance (e.g., Medicaid) | 11 (14) |
| Place where household members seek medical care^b^ |  |
| Private doctor | 44 (54) |
| Emergency department or urgent care center | 26 (32) |
| Free clinic | 15 (19) |
| Hospital | 14 (17) |
| Company doctor | 1 (1) |
| Do not use any medical care | 4 (5) |
| Household member admitted to hospital in past 3 months^b^ | 9 (11) |
| ^a^Totals for each characteristic may not sum to the total number of households due to missing information. | |
| ^b^Totals do not sum to 100% because participants could report more than one of the categories. | |
