# Supplementary material for: Livestock-Associated, Antibiotic-Resistant Staphylococcus aureus Nasal Carriage and Recent Skin and Soft Tissue Infection among Industrial Hog Operation Workers
Source: PLoS One. 2016 Nov 16;11(11):e0165713. doi: 10.1371/journal.pone.0165713 (PMC5112983; doi:10.1371/journal.pone.0165713)
Supplement: S3 Table — (DOCX) [file pone.0165713.s004.docx]

S3 Table. Antibiotic resistance patterns of *S. aureus* recovered from the anterior nares of industrial hog operation workers and household members in North Carolina, 2013-2014^,b^

|  | Number of participants carrying isolate with resistance pattern | |
| --- | --- | --- |
| Resistance pattern | Workers  N=103 (%) | Household members  N=80 (%) |
| β-lactams | 17 (17) | 17 (21) |
| Fluoroquinolones | 1 (1) | 0 |
| β-lactams, aminoglycosides | 0 | 1 (1) |
| β-lactams, tetracyclines | 4 (4) | 2 (3) |
| β-lactams, lincosamides, macrolides | 6 (6) | 5 (6) |
| β-lactams, cephalosporins, tetracyclines | 1 (1) | 0 |
| β-lactams, aminoglycosides, lincosamides, macrolides | 1 (1) | 0 |
| β-lactams, fluoroquinolones, lincosamides, macrolides | 1 (1) | 0 |
| β-lactams, tetracyclines, lincosamides, macrolides | 8 (8) | 3 (4) |
| β-lactams, tetracyclines, lincosamides, macrolides, aminoglycosides | 2 (2) | 0 |
| β-lactams, tetracyclines, lincosamides, macrolides, streptogramins | 2 (2) | 0 |

^a^Isolates resistant to three or more class of antibiotics were defined as multidrug-resistant *S. aureus* (MDRSA).

^b^A list of antibiotics tested is presented by class in S1 Table.
