# Supplementary material for: Livestock-Associated, Antibiotic-Resistant Staphylococcus aureus Nasal Carriage and Recent Skin and Soft Tissue Infection among Industrial Hog Operation Workers
Source: PLoS One. 2016 Nov 16;11(11):e0165713. doi: 10.1371/journal.pone.0165713 (PMC5112983; doi:10.1371/journal.pone.0165713)
Supplement: S4 Table — (DOCX) [file pone.0165713.s005.docx]

S4 Table. Summary of industrial hog operation worker exposures and recent SSTI in past three months in North Carolina, 2013-2014.

|  | Total | SSTI^c^ |
| --- | --- | --- |
|  | N^a^ (%) | N (%) |
| Number of participating workers | 103 (100) | 6 (100) |
| Years employed at current hog operation |  |  |
| <1 | 21 (11) | 2 (33) |
| 1-5 | 44 (43) | 0 (0) |
| 6-9 | 11 (11) | 2 (33) |
| ≥10 | 27 (26) | 2 (33) |
| Average hours/week |  |  |
| ≤40 | 19 (19) | 0 (0) |
| 41-50 | 31 (31) | 3 (50) |
| 51-60 | 40 (40) | 3 (50) |
| >60 | 9 (9) | 0 (0) |
| Average number of hogs in contact with per day |  |  |
| ≤1000 | 64 (67) | 4 (67) |
| 1001-5000 | 22 (23) | 2 (33) |
| >5000 | 10 (10) | 0 (0) |
| Life stage of hogs in contact with at work^b^ |  |  |
| Sows/farrow piglets/weaned/nursery | 70 (68) | 5 (83) |
| Feeder/finish | 29 (28) | 1 (17) |
| Direct contact with animals other than hogs at work | 8 (8) | 1 (17) |
| Work with breeding pigs | 24 (23) | 4 (67) |
| Draw or collects blood from hogs | 9 (9) | 1 (17) |
| Give hogs shots | 70 (70) | 6 (100) |
| Handle dead hogs | 79 (79) | 5 (83) |
| Eat at hog operation | 89 (89) | 6 (100) |
| Use of face mask at work |  |  |
| Always | 37 (37) | 0 (0) |
| Sometimes | 45 (45) | 4 (67) |
| Never | 18 (18) | 2 (33) |
| Use of other personal protective equipment at work |  |  |
| Always wear gloves | 86 (86) | 5 (83) |
| Always wear long sleeves and pants, or coveralls | 86 (86) | 4 (67) |
| Always wear boots or other foot protection | 95 (96) | 6 (100) |

^a^Totals for each characteristic may not sum to the total number of workers due to missing information.

^b^Totals do not sum to 100% because participants could report more than one of the categories.

^c^Comprises individuals who reported “Yes, in the past three months” to any of the following: *S. aureus* infection; skin boil; pus-filled abscess; red, painful, swollen skin bump or “pimple”; or spider bite that is itchy. Participants were shown pictures of *S. aureus* infections with each of these presentations prior to answering this question.
