# Supplementary material for: Livestock-Associated, Antibiotic-Resistant Staphylococcus aureus Nasal Carriage and Recent Skin and Soft Tissue Infection among Industrial Hog Operation Workers
Source: PLoS One. 2016 Nov 16;11(11):e0165713. doi: 10.1371/journal.pone.0165713 (PMC5112983; doi:10.1371/journal.pone.0165713)
Supplement: S5 Table — (DOCX) [file pone.0165713.s006.docx]

S5 Table. Distribution of *S. aureus spa* types recovered from the anterior nares of industrial hog operation workers and household members in North Carolina, 2013-2014.

| *spa* type | Overall^a^ | Workers | Adult  household members | Minor  household members^a^ |
| --- | --- | --- | --- | --- |
|  | N=75 (%) | N=45 (%) | N=6 (%) | N=25 (%) |
| t002 | 1 (1) | 1 (2) | 0 | 0 |
| t008 | 3 (4) | 3 (7) | 0 | 0 |
| t018 | 1 (1) | 1 (2) | 0 | 0 |
| t021 | 1 (1) | 0 | 0 | 1 (4) |
| t031 | 2 (3) | 1 (2) | 0 | 1 (4) |
| t034^b^ | 5 (7) | 2 (4) | 2 (33) | 1 (4) |
| t065 | 4 (5) | 3 (7) | 0 | 1 (4) |
| t089 | 1 (1) | 0 | 0 | 1 (4) |
| t091 | 3 (4) | 1 (2) | 0 | 2 (8) |
| t094 | 2 (3) | 2 (4) | 0 | 0 |
| t10119 | 1 (1) | 0 | 0 | 1 (4) |
| t1430^c^ | 1 (1) | 1 (2) | 0 | 0 |
| t1446^b^ | 1 (1) | 1 (2) | 0 | 0 |
| t148 | 2 (3) | 0 | 0 | 2 (8) |
| t150 | 2 (3) | 2 (4) | 0 | 0 |
| t189 | 2 (3) | 1 (2) | 0 | 1 (4) |
| t230^b^ | 1 (1) | 0 | 0 | 1 (4) |
| t233 | 4 (5) | 2 (4) | 0 | 2 (8) |
| t2868 | 1 (1) | 0 | 0 | 1 (4) |
| t3270^c^ | 1 (1) | 1 (2) | 0 | 0 |
| t337^c^ | 6 (8) | 6 (13) | 0 | 0 |
| t3446^c^ | 1 (1) | 1 (2) | 0 | 0 |
| t346 | 1 (1) | 0 | 0 | 1 (4) |
| t3802 | 1 (1) | 0 | 1 (17) | 0 |
| t4976 | 3 (4) | 1 (2) | 0 | 2 (8) |
| t571^b^ | 2 (3) | 2 (4) | 0 | 0 |
| t5739 | 2 (3) | 0 | 0 | 2 (8) |
| t5883^b^ | 1 (1) | 1 (2) | 0 | 0 |
| t616 | 2 (3) | 2 (4) | 0 | 0 |
| t622 | 1 (1) | 1 (2) | 0 | 0 |
| t6228 | 1 (1) | 1 (2) | 0 | 0 |
| t645 | 2 (3) | 1 (2) | 0 | 1 (4) |
| t659 | 2 (3) | 1 (2) | 1 (17) | 0 |
| t688 | 1 (1) | 0 | 0 | 1 (4) |
| t692 | 3 (4) | 3 (7) | 0 | 0 |
| t701 | 3 (4) | 2 (4) | 1 (17) | 0 |
| t7226 | 4 (5) | 1 (2) | 0 | 3 (12) |

^a^Totals are one less than previously presented as one *S. aureus* isolate from an adult is currently undergoing genotyping.

^b^Associated with *S. aureus* CC398 (an indicator of livestock association).

^c^Associated with *S. aureus* CC9 (an indicator of livestock association).
