# Supplementary material for: Livestock-Associated, Antibiotic-Resistant Staphylococcus aureus Nasal Carriage and Recent Skin and Soft Tissue Infection among Industrial Hog Operation Workers
Source: PLoS One. 2016 Nov 16;11(11):e0165713. doi: 10.1371/journal.pone.0165713 (PMC5112983; doi:10.1371/journal.pone.0165713)
Supplement: S6 Table — (DOCX) [file pone.0165713.s007.docx]

S6 Table. Characteristics of *S. aureus spa* types shared within participant households in North Carolina, 2013-2014.^a^

| Household | Participant type | *spa* type | Characteristics of livestock association | | | |
| --- | --- | --- | --- | --- | --- | --- |
|  |  |  | *scn*-negative | tet-resistant | CC398 | CC9 |
| A | Worker^b^ | t4976 |  | **x** |  |  |
|  | Minor |  |  |  |  |  |
| B | Worker | t701 |  | **x** |  |  |
|  | Adult household member |  |  | **x** |  |  |
| C | Worker | t645 |  |  |  |  |
|  | Minor |  |  |  |  |  |
| D | Worker | t7226 |  |  |  |  |
|  | Minor |  |  |  |  |  |
| E | Worker | t233 |  |  |  |  |
|  | Minor |  |  |  |  |  |
| F | Worker^b^ | t659 |  |  |  |  |
|  | Adult household member^b^ |  |  |  |  |  |
| G | Worker | t233 |  |  |  |  |
|  | Minor |  |  |  |  |  |
| H | Worker | t337 | **x** | **x** |  | **x** |
|  | Worker |  | **x** |  |  | **x** |
| I | Worker | t094 |  |  |  |  |
|  | Worker |  |  |  |  |  |
| J | Minor | t5739 |  |  |  |  |
|  | Minor |  |  |  |  |  |

^a^Fifty-nine of 81 participating households contained more than one study participant. Ten of these 59 households contained two individuals who carried the same *S. aureus spa* type at baseline.

^b^Carried multidrug-resistant *S. aureus* (MDRSA).
